# Certified Peer Support in the Field of Homelessness: Stories Behind the Work

**DOI:** 10.1007/s10597-024-01315-1

**Published:** 2024-07-02

**Authors:** Margriet de Zeeuw Wright, Candice Morgan

**Affiliations:** College of Social Work, University of South Carolina, Columbia, USA

**Keywords:** Peer support, Certified peer support, Homelessness, Housing, Thematic analysis, Qualitative research

## Abstract

Certified peer support specialists (CPSS) are used as a paraprofessional workforce to engage hard-to-reach populations, including people experiencing homelessness. Thematic analysis was used to explore with CPSS (N = 7) what contributed to their effectiveness when working with this population. Participants were recruited at a HUD lead organization in the southeastern United States. Open-ended semi-structured questions were used in online, synchronous interviews. Themes related to three areas, experience, competence, and the organization, contributed to participants being effective. Specifically, interviewees observed that their lived experiences and abilities to speak a common language with clients contributed to their effectiveness. They identified how personal qualities and unique skillsets suited them for the work. Participants also valued the training they received; certification helped them to develop competencies and to balance vulnerability, empathy, and connection. Finally, participants attributed their effectiveness to clarity about their roles within the organization, supervision, attention to self-care, and co-worker support. Findings from this study may have implications for the value of lived and learned knowledge coexisting in organizations serving those who experience homelessness.

## Introduction

An individual who lacks a fixed, regular, and adequate nighttime residence may be defined as homeless (de Sousa et al., 2023). A recent Point-in-Time count in the United States found that approximately 653,000 people were experiencing homelessness (de Sousa et al., 2023). These data reflect an increase of 12%. Six in ten people were experiencing sheltered homelessness, meaning they resided in emergency shelters, transitional housing, or safe havens. Four in ten people were experiencing unsheltered homelessness, meaning they lived in places not meant for human habitation (de Sousa et al., 2023). Given its rise, homelessness is a social problem requiring immediate attention.

Homelessness may lead to losses in health, safety, and connectedness. People experiencing homelessness are at risk for acute and chronic health issues (Hwang et al., 2011; Krakowsky et al., 2013; Raoult et al., 2001; Wadhera et al., 2020). High rates exist for mental illness (Gutwinski et al., 2021), cognitive impairment (Stergiopoulos et al., 2015), and drug and alcohol use (Aubry et al., 2012). Safety is at risk, hope diminishes, and trust in support services erodes. The result can be isolation and feelings of worthlessness, leading to depression and loneliness (Sanders & Brown, 2015). This population has been described as hard to reach (Miler et al., 2020) and given the complex, multidimensional aspects of being unsheltered and having substance use and / or health and mental health issues, recovery is difficult to achieve.

### Recovery

It should be noted that “recovery from” is different from being “in recovery.” People may recover from a broken leg, while people who struggle to exit homelessness and overcome substance use or mental health issues, may be in recovery for a lifetime. Kelly and Hoeppner (2015) sought to clarify recovery as a construct and proposed a bi-axial formulation of recovery for substance use relevant for this discussion of homelessness. According to the authors (Kelly & Hoeppner, 2015), there is a reciprocal relationship between remission of a substance use disorder on the one hand, and recovery capital on the other. Recovery capital includes physical and mental health, housing, social relations, education and employment, and meaning and purpose in life (White & Cloud, 2008). The bi-axial formulation places remission on one axis and recovery capital on the other (Kelly & Hoeppner, 2015). The model proposes that as addiction remission becomes more stable and prolonged, the positive benefits of recovery capital become more evident. From this viewpoint, “recovery is a dynamic process characterized by increasingly stable remission resulting in and supported by increased recovery capital and enhanced quality of life” (Kelly & Hoeppner, 2015, p. 179).

Kelly and Hoeppner (2015) suggest that the relationship between remission and recovery capital is mediated by increased coping abilities and decreased experiences of stress. The authors use Lazarus and Folkman’s (1984) Transactional Model of Stress and Coping to conceptualize these mediating elements. The model suggests that individuals engage in appraisals in the face of stress. A primary appraisal determines if a situation warrants attention. If it is assessed as stressful, a secondary appraisal determines whether the stress exceeds resources and requires strengthened coping. For individuals early in their recovery process, the stress response is more sensitive and may interfere with new learning and increase the risk of relapse (Kelly & Yeterian, 2013). Simply stated, managing substance use and accumulating recovery capital hinge on stress appraisal and increased coping; many who succeed do so through the support of their peers.

### Peer Support

The history of peer support dates back decades, however, it is best known in the form of the twelve-step model (Alcoholics Anonymous, 2002), where sponsors mentor peers who are managing alcohol use. Howard “Howie the Harp” Geld (Harp & Zinman, 1994) was a consumer and consumer rights activist of the 1970’s who believed that “to transform the treatment system, you need to transform its workforce” (Community Access Inc., 2020 para.2) and that “those closest to the problem have a unique part to play in shaping the solution” (Community Access Inc., 2020 para.3). This is supported by a recent report (National Institute for Health and Care Excellence, 2022) that recommended that peer workers with lived experience of homelessness be involved in the design and delivery of services, to improve the quality of health and social care to this population. Peer support has grown and developed into a managed resource. As of 2023, 49 of 50 states in America have established programs to train and certify peer support workers^1^ (SAMHSA, 2023). Peers assist others with similar challenges, often related to mental health and substance use issues; however, peers support others “in a wide range of nonclinical activities, including advocacy, navigation and linkage to resources, sharing of experience, social support, community and relationship building, group facilitation, skill building, mentoring, goal setting, and more” (SAMHSA, 2023, p. 7). Training and certification “attest that an individual has the skills and knowledge required for the peer support services profession” (SAMHSA, 2023, p. 7).

Chinman et al. (2016) described a wide range of services and activities provided by peer support workers that include “promoting hope, socialization, recovery, self-advocacy and developing natural supports and community living skills” (p. 2). There is consensus that empathy and respect, a nonjudgmental stance, voluntary participation, direct communication, mutual responsibility, shared power, and reciprocal support guide peer support (National Association of State Mental Health Program Directors, n.d.). SAMHSA (2018) has identified an emerging set of core competencies of peer support workers including: engaging in collaborative and caring relationships; offering not simply support but personalized support; sharing lived experiences of recovery; recovery planning; linking to resources, services and supports; providing information about skills; managing crises; valuing communication; engaging in collegial collaboration and teamwork; promoting leadership and advocacy; and promoting growth and development. Barker and Maguire (2017) reported that peer support effectively contributed to shared experiences, role modelling, and social support.

Valuing the contribution that peer support workers offer means valuing a non-professional vantage point (Mead & MacNeil, 2006). Perceived empathy is one of the key benefits of peer support (Campbell & Leaver, 2003). Clay (2005) described this empathy and respect in terms of safety and acceptance with a focus on an individual’s strengths, noting that the mutual sharing of stories provided encouragement and an opportunity to offer technical support to peers. In this respect, peer support workers function as role models to show that recovery is possible (Clay, 2005). The experience of simultaneously being in recovery while assisting others in recovery has proven to be mutually beneficial; however, there are also vulnerabilities contained within this type of relationship that are worth considering. Challenges can be conceptualized as occurring at an individual level and at a system level. Dennis (2003) identified peer worker stress and over-involvement as potential individual-level challenges. There is also the possibility that peer support workers, in acting as role models, might model negative behaviors (Barker et al., 2019). At a system-level, Yuen and Fossey (2003) noted workload demands as something that required attention. Mowbray et al. (1998) suggested that professional colleagues may not value the recovery messages offered by peer support workers and may undermine the efforts and confidence of their peers. In their comprehensive review of the literature, Miler et al. (2020) supported these points and identified five key challenges for peer support workers; this included vulnerability; authenticity; boundaries; stigma; and a lack of recognition of the value of peers. Vulnerability was framed in terms of threats to peer support workers’ own recovery. Maintaining authenticity was a challenge for some peer workers who felt distanced the longer they were in recovery or the more regulated they were as employees. Boundaries were conceptualized as a balance of rapport and self-disclosure, with some limits. Stigma was framed in the literature in a couple of ways, including workplace experiences of discrimination and peer workers stigmatizing their own community. Valuing peer support workers was discussed in terms of respect for the work and adequate compensation. Miler et al. (2020) recognized the challenging work done by peer support workers and offered guidelines for how this workforce can be integrated effectively into service delivery for those experiencing homelessness. Their review is especially relevant to this study, as it targeted peer support in homelessness.

### Focus and Aims of the Study

This pilot study took place with an organization whose mission was to break the cycles of homelessness. The organization was a coordinated entry point serving 13 counties in the southeast region of the United States. Four to five of these counties consistently ranked the lowest on national health outcomes, income, and housing indicators (personal communication A.G.). People with disabilities, veterans, youth, families, and survivors of intimate partner violence were served by the organization. All clients were in housing crises, and depending on their needs, they could also address substance use issues in transitional housing, receive permanent supportive housing assistance, case management, claims support, and homelessness prevention services. The organization grew exponentially in recent years from an annual budget of $140,000 and one full-time employee, to a $10 million annual budget and 54 employees. Organizational funding sources included the U.S. Department of Housing and Urban Development (HUD), the U.S. Department of Veterans Affairs (VA), and private money. Half of the program’s direct service employees, who were called “Team Members,” had lived experiences of homelessness and substance use, and / or mental health issues. Their roles related to intake, housing support, case management, and outreach. These peer support workers inspired and contributed to this study. The study’s aim was to explore what contributes to the effectiveness of Certified Peer Support Specialists (CPSS) in the field of homelessness.

## Methods

CPSS were involved in the study design and recruitment, data collection, analysis, and reporting. Approval was obtained from the Institutional Review Board (IRB) of the University of South Carolina. Open-ended semi-structured interviews were conducted. An interview guide grew out of Miler’s et al. (2020) systematic review of peer support at the intersection of homelessness and problem substance use. The authors proposed guidelines for how to embed peer workers in services. Furthering their work, this study asked participants to discuss whether and / or how any of the following elements contributed to their effectiveness: (1) the clarity of their role description, (2) the adequacy of their compensation, (3) the degree of support they received, (4) the level of training and development and advancement opportunities, (5) perceptions of their value within the organization, and (6) the degree of flexibility around workplace accommodations, if needed. This qualitative study used a thematic analysis approach, including constructionist, inductive, and latent methods (Boyatzis, 1998; Braun & Clark, 2006; Burr, 1995; Hayes, 1997; Rubin & Babbie, 2016), to discern and present insights.

### Sampling, Study Design, Recruitment, and Data Collection

Seven of a possible 13 CPSS participated in the study. CPSS were recruited through an online information session, multiple emails, and flyers placed at the organization. Participants were required to have received certification as CPSS and to work as CPSS at the time. The seven participants were between the ages of 29 and 42 (mean age 35.3). Of these, five identified as female and two as male. While participants were given the choice whether to self-disclose, 6 explicitly identified that they had experienced homelessness. One reported a history of mental health issues in addition to homelessness. All seven participants reported lived experiences of addiction. Time in recovery ranged from 5 to 16 years (mean time in recovery 9.42 years). Job tenure at the agency varied from 9 months to 5.5 years (mean job tenure 2.5 years). Two longer-serving participants reported earning internal promotions, leading to greater responsibility.

All participants had opportunities to ask questions about the study and gave informed consent to participate. Participants also consented to the publication of results. They were interviewed on average for an hour. Interviews occurred through an online platform and were video and audio recorded. Those who agreed to participate received $20 for completing the interview. We developed a clear policy on payment and recognition prior to involvement. We disclosed that each person would receive $20 cash in the mail to an address of their choosing. The $20 was mailed in a thank you card, detailing why the recipient was receiving the money and thanking them for their time, within a week following the interview. With respect to recognition, we chose to keep the identities of the participants confidential to encourage interviewees currently employed in the agency to be open and honest about their experiences there. We involved one key informant in the decision to provide $20. We sought advice from a supervisor who confirmed this would be comparable to the typical hourly wage at the agency. We also arranged for participants to be allowed to complete the interview while on the clock at the agency, hence receiving double compensation. Participants did not have to take time off to participate.

### Data Analysis

Thematic analysis was used to identify, analyze, and report patterns with the data without being tied to any pre-existing theoretical framework (Braun & Clark, 2006). Both MW and CM were responsible for the coding. We relied on a constructionist method, “which examines the ways in which events, realities, meanings, experiences, and so on are the effects of a range of discourses operating within society” (Braun & Clark, 2006, p. 81). We understood that our participants’ experiences and internal constructions of reality form in discourse, which is an understanding held with a social constructionist approach (Gergen, 1999). As we conducted and analyzed interviews, we paid close attention to how participants recalled experiences as CPSS and how those recalled experiences formed a reality for them.

An inductive approach was taken to identify themes and patterns. Our goal was to provide a detailed analysis of some aspects of the data, namely what contributes to the effectiveness of CPSS, according to self-report. Focusing on role description, compensation, support, development, value, and accommodations (Miler et al., 2020), we took a reflective thematic analysis approach (Braun & Clark, 2006). We considered the interview data, as well as our own positionality as social workers and the context of CPSS work within an organization. We chose to identify latent themes and patterns within the data (Boyatzis, 1998) ascribed by the participants, as well as by our interpretations. Thematic analysis at the latent level was a good fit with the constructionist approach (Burr, 1995).

Thematic analysis was conducted in six phases as depicted in Table 1. CM and MW worked through the phases separately first, and then together meeting weekly to compare, discuss, and focus our analysis. We strove for consensus rather than compromise during data analysis. To confirm the credibility of our results, we member checked (Birt et al., 2016) our data analysis with several participants for accuracy, and resonance with their experiences.

**Table 1:** Six thematic analysis phase descriptions (Braun & Clark, 2006)

Phase	Description
1	Goal: Familiarize ourselves with the data Process: Repeated reading of the transcripts and viewing video recordings
2	Goal: Produce initial codes Process: Worked through entire data set, coded for potential themes, organized data extracts and codes
3	Goal: Sort and collate coded data into identified themes Process: Sorted different codes into potential themes and collated relevant coded data extracts from both coders, bringing both coders’ sets of codes together for sorting
4	Goal: Review themes; create a thematic map (see Fig. 1) Process: Reviewed themes, deleted sub-themes with little data to support, collapsed multiple sub-themes into one
5	Goal: Define and redefine themes destined for findings section Process: Reviewed themes in the thematic map to determine if they captured the essence of the stories and answered the research question
6	Goal: Write findings section Process: Composed stories from the data, wove examples into the analytic narrative, member checked

**Fig. 1:**
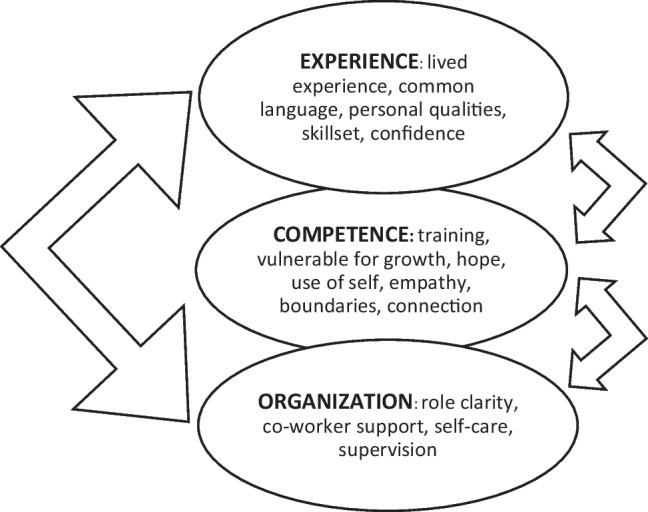
Phase 4 Thematic Map in Response to the Question: What Contributes to the Effectiveness of CPSS in the Field of Homelessness?

## Results

The study found that themes broadly related to three areas, namely experience, competence, and the organization, contributed to participants being effective in their jobs. First, their lived experiences and abilities to speak a common language with clients contributed to their effectiveness. They identified that their personal qualities and unique skillset gave them confidence to do the work. Second, participants valued the training they received, as certification helped them to understand and develop competencies to use themselves to offer hope to clients, and to balance vulnerability, empathy, and connection with boundaries. Third, participants in this study spoke about how the organization supported them to be effective in their positions as peer workers. Formally, clarity about their roles, supervision, and attention to self-care were important. Informally, co-worker support was noted as vital to their ability to do the work effectively. There may be reciprocal interaction among factors that made *these participants with these competencies at this organization* effective in their roles.

### Experience

Several themes emerged that described what drew these participants to this work. These themes related to their lived experiences of homelessness co-occurring with either substance use or mental health issues, common language, a unique skill set, and confidence in discerning client needs. Nearly all participants explicitly stated that working hard was a part of who they were. They were passionate about their work and internally motivated to not only work at, but work for, the organization.

### Lived Experiences Compared with Learned Knowledge

These participants recognized the value of their lived experiences. Participants saw the empathy, skill, and knowledge their co-workers brought to the work. This challenged previously held beliefs for some, who thought that helpers had to have “been there” to appreciate the realities of their experiences of homelessness, mental health issues, or substance use.

And that was probably one of the things I was closed minded to during CPSS (training). I remember going to a rehab and the counselor not being an addict and I immediately had shut down. I didn't want to talk to her. She doesn’t know how I feel. (Participant #2)

Another participant suggested there was a difference between lived and learned knowledge. There was a sense that workers without lived experience could be receptive while those with lived experiences could be perceptive. For participants in substance use recovery, however, the day-to-day work could trigger reminders of their own recovery journeys. For this reason, participants noted that some peer workers return to use or leave their positions.

I think it may be a little more challenging. 'cause it's more … book learned, you know or taught in a training or but it's something very different to know it and genuinely practice it. … There have definitely been times where they'll hand me a nice slice of truth and reality and recovery all at the same time. That'll punch me right in my gut. (Participant #7)

Participants saw the benefits of both lived and learned knowledge being used in services to clients. Some reported that they struggled to understand the professional language of book knowledge but recognized that they contributed important understandings of client language.

### Common Language

Participants clearly identified with their clients and noted their skills for connecting with this hard-to-reach population. They spoke a common language, they knew how to complete housing applications because they, themselves, had done so in the past. They understood barriers to being housed and the frustrations associated with engaging with services. Participants knew the community and the experiences intimately because they were part of it.

You know, if you've been out there on the streets for over a year too, you know, you've seen some things you've done some things. … I can’t plant the desire to change, I can only show what change looks like. They have somebody walking right alongside them. … I'm not coming from a place of judgment. I'm trying to come from a place of compassion and understanding. (Participant #5)

That experience is going to inform how you talk to others, how you treat others. (Participant #1)

Participants spoke and understood clients’ language. Because they had “been there,” they came from a place of acceptance and compassion, knowing how important this was in their own experiences. The work was meaningful for them.

### Personal Qualities, Skillset, and Confidence

These participants found meaning and purpose in their work. Some identified formerly being in jobs with little stimulation and no chance for growth or promotion. Nearly all the participants spoke of their strong work ethic. Because they had worked hard to overcome personal challenges, they were willing to work hard to maintain their growth. They expressed gratitude that the unique skills they possessed, due to their life experiences, could be used to benefit their communities.

So, learning along the way and making a whole bunch of errors and then figuring out how to do it, and you know, it all lined up the way it needed to and for whatever reason the skill set I have was unique for this job and it seemed to be a nice fit. (Participant #6)

Participants expressed confidence in their discernment. They recounted experiences with clients that required judgment, such as when to bend the rules, and how to identify the urgency of some moments for clients to capitalize on their readiness to change. Their instincts and lived experiences enabled them to recognize these moments as client opportunity or opportunism.

OK that’s a crisis … versus an emergency…. Gut instinct… having been a person who manipulated the system, you can tell. Nails done but not paying rent. Lack know-how to budget… Some of this can’t be taught… what works for me is that connection. I’m learning the “correct” language. A lot of it is instinctual. (Participant #5)

These participants that stepped forward to give back to their community, could channel their energy in a meaningful and purpose-filled way. This enhanced their own lives. They saw value in their unique skill set, could speak the language of those they served and had confidence in their ability to discern the needs of clients. This confidence may have come from their own lived experiences, or it may have come from their certification. We turn now to the role of training in participants’ effectiveness.

## Competence

Participants explicitly discussed their CPSS training in our conversations about their effectiveness. State certification^2^ was a requirement for their roles, to ensure competence to protect vulnerable populations. Training, for these participants, tapped into a willingness and ability to be vulnerable for growth. Participants made decisions about when, or if, to share their own stories with clients. Themes emerged related to hope, use of self in service to others, empathy, and finally the importance of both boundaries and connections.

### Vulnerable for Growth

Most participants spoke of their professional growth and development. Growth was often coupled with a willingness to be vulnerable and to ask for help. They identified the learning curve required to be CPSS and were open to seeking and receiving assistance.

If I'm going to work with them and if I can't, then I'm not afraid to cry mercy and ask for some help with some capacity. (Participant #6)

Vulnerability was self-evident for these participants, who implicitly or explicitly linked this to their lived experiences. A sense of pride in their accomplishments was also evident. Participants spoke of desires for promotion and advancement which for almost all of them was unimaginable at some point in their lives. They were hopeful and sought to be a source of hope for their clients.

### Hope

Many participants voiced gratitude that they had experienced others in their lives who had been further along on the path in some type of recovery (housing, substance use, mental health). They understood firsthand the benefits of peer support. Seeing this had given them some hope, and this was their chance to give that back in some way. Participants wanted clients to know that they would not give up on them and that housing stability was possible.

I went through a long-term place, and it was the women that were further along in the path that I've seen. They were happy and they were truly smiling, and it made all the world to me to see it working for other people. … I give a little hope… I guess I would go back to it being the hope, not ever wanting someone to feel like people had given up on them, or that there's no chance for them. (Participant #2)

Hearing participants speak of their lived experiences, their desire for training, and their willingness to be vulnerable in growth and development; it was clear they had the capacity for self-reflection. Their responses to the study’s questions were honest and introspective.

### Use of Self in Service to Others

A significant part of peer support depends on self-disclosure. Participants were mindful that their role was to support clients rather than get their own needs met. They reported being especially effective at engaging clients and getting their buy-in for change. While they acknowledged that sharing their lived experiences and stories of homelessness with clients could be beneficial for themselves, they were clear that their attention needed to focus on the clients they served.

I think it's heavy handed when I'm just like hey, I'm you know I've been homeless before too. Like I said, I only pull that out when it when it feels right, like when it feels like it serves a purpose. When I feel like it will help them internalize some of the things I've said. And then from there you have your buy-in. Now … we're walking right beside each other on a mission and on a journey. (Participant #6)

Determining when to self-disclose and share their lived experiences required participants to effectively read the situation and their clients. This ability revealed participants’ empathy.

### Empathy

Participants demonstrated empathy for their clients. They recalled the challenges and barriers they had faced in seeking and retaining housing or in managing substance use or mental health. Participants acknowledged that training helped open their minds to the idea that there were many paths on a client’s journey. Especially for participants in substance use recovery, training helped to soften the edges on their individual opinions about what clients should be doing.

We just had the lady up there who just got housed yesterday. She hasn't even been in there 12 hours yet and she's really panicked. And you know, a lot of people don't understand that she may not be ready for that right now. She just may not be, you know. (Participant #7)

12-steps taught me absolutes. Peer support is more lenient, meeting you where you’re at versus this is the way you do it. Peer support is, “this is the experience I have, but let’s talk about you though.” (Participant #5)

Empathy came with a risk for overidentification. Participants could see themselves in the lives of clients and while that connection was the strength of their role, there was also the need for boundaries.

### Connections and Boundaries

Themes of boundaries and connections were prevalent in interviews. The importance of connection showed up in the meaning participants derived from their work. It was evident in their willingness to be vulnerable and grow. Connections were foundational to participants’ empathy and engagement with those they served. The flipside of connections were boundaries. All participants recognized the importance of boundaries in their effectiveness. Some participants noted that they developed boundaries over time either because their instinct was to save clients and they realized this was neither possible nor their role; or because their boundaries were violated, and they needed to shore them up. Any worker in human services likely understands this. What may be unique to peer support is how to maintain boundaries in a role that depends on personal self-disclosures and identification with clients. An additional complicating factor surfaced when they knew clients from their previous lives.

It's a very fine line to walk… There's not even a whiff of impropriety. And you know, in the job interview they brought up hey, if you know you're going to get approached by people in the rooms about going to the front of the line. And I've had people. I mean my first meeting after getting the job I had someone come up to me and say hey, like I'm on the list. And I’m like, oh man, I know where you live, I can see your house from here, you're not homeless. (Participant #4)

It was evident in interviews with participants that their training contributed to their effectiveness. They presented themselves as lifelong learners. They believed in the power of peer support; some having experienced it themselves. They instilled hope. They showed a willingness to be vulnerable for growth. They demonstrated skills for self-reflection, empathy, and considering boundaries. Their focus was squarely on connection in human relationships and on the needs of their clients. All of this reflects the core competencies of peer support (SAMHSA, 2018). Completing the training and being certified did not guarantee longevity or success as a peer support worker. These participants talked about the significant role of the organization in their effectiveness.

## Organization

Participants identified the importance of the organization in supporting their effectiveness with clients. Themes emerged related to the clarity of their role within the organization, the value of co-worker support, the promotion of self-care, and regular supervision. These elements of the organization were also highlighted as protective factors against turnover.

### Role

Participants spoke about the positive culture of the organization in terms of shared mission and values. They reportedly were valued for their contributions and supported in active engagement. Many participants framed this in terms of belonging, and a family atmosphere, which in this case, led to familiarity and encouragement. Circling back to the unique skill set possessed by these participants, they reported feeling valued (as some were handpicked from the community and asked to consider positions with the organization) and in turn, they valued being in a place where they could be themselves. Their role within the organization was clear, and they did not have to “pretend” to be something they were not.

When I first started here, I was enveloped … I didn't have to conform to a certain ideology that people have when others work for nonprofits. Every single person here is different… And through that uniqueness, it's just this crazy big family and we all offer something unique. (Participant #1)

Being able to show up as their authentic selves contributed to participant self-efficacy. This confidence and competence increased with the support of their co-workers.

### Co-worker Support

The skills participants learned in their training such as collaboration, valuing relationships, and communication were reportedly as useful in their interactions with co-workers as they were with clients. Listening skills, empathy, affirmations, and tangible support were reportedly given and received and were viewed as essential ingredients in participants doing their jobs well. Co-worker support was seen as a significant protective factor against turnover.

My coworkers are by my side. People come, you know. I've helped them just as much as they've helped me. We have a very give and take relationship, and that's really across the board now. (Participant #6)

Given the nature of the work, worker turnover was a reality. Participants spoke of risks like burnout or return to use for those in substance use recovery. One participant framed turnover in terms of amateur versus mature recovery. There were some workers who were still journeying on the path towards being ready and able to help others. Not being ready to shoulder the responsibilities of peer support was identified as a barrier to effectiveness.

I did not know how to handle my emotions. Did not know how to be unselfish. I really had no idea what I was doing… And so, that's what I mean by amateur or not ready recovery. (Participant #7)

Organizational administration seemed to recognize the risks of burnout and turnover and offered formal and informal self-care strategies to employees.

### Self-care and Supervision

Participants discussed ways the organization supported their self-care. These included informal measures like celebrating milestone anniversaries, encouraging mental health days, time-off to attend the funerals or celebrations of life of clients, on-site book clubs, and yoga, and formal measures like ongoing training and supervision.

(Pay) could be better but self-care perks you can’t put a dollar amount on. (Participant #5)

Participants engaged in regular supervision with a licensed social work supervisor. Several participants reportedly sought and earned promotions within the organization. One participant had the insight that several workers currently in leadership positions at the organization had lived experiences themselves. This offered hope that further advancement was possible. This begs the question whether workers who have “been there” had sought promotion and taken their training and recovery principles with them in a bottom-up movement, or whether there was a conscious top-down promotion of peer worker skillsets and shared values. Regardless, participants appreciated being part of the organization and experiencing the support they received from across the organization to do their jobs well.

(D)efinitely one of our strong suits is the bond that we all share… seeing it, it makes you want to do it, and that's the butterfly effect throughout the organization. …I could think of (the E.D.) doing it for random staff members, random staff members doing it across departments. So yeah, it's pretty ubiquitous. (Participant #4)

Participants identified the importance of the organization in supporting their effectiveness. Themes emerged related to having a clearly identified role, co-worker support, regular supervision, and organization-supported self-care. Participants saw these efforts as protective factors against burnout and turnover and necessary for promotion and advancement. There seemed to be a good fit between these participants with their lived experiences and unique skill sets, the competencies they learned through certification as peer support workers, and the supports found in the organization.

## Discussion

… if you can see it, you can be it.

Homelessness is a complex problem that requires innovative social solutions. One approach taken by the organization in this study was to employ CPSS in their workforce. The question posed in this study was not whether CPSS are effective in their role. Instead, this study explored what contributes to CPSS effectiveness when working with those experiencing homelessness. We asked the people doing the work to self-report on their effectiveness. We will address the limitations of this method in a moment; however, asking CPSS to reflect on the work they did, honored, and included their perspectives in a co-created understanding. Participants in this study saw their work as a way to give back to their community in a meaningful and purpose-filled way. They recognized the value of their unique skill set to reach those experiencing homelessness, they could speak the language of those they served, and had confidence in their ability to discern the needs of clients. These skills contributed to rapport and enabled them to actively engage with the population they served. This is important given how difficult it can be to reach those experiencing homelessness. Not all CPSS workers were ready to assume the role, and some needed time to grow into it. For participants in this study, they made a commitment to seek training and certification, and this clearly contributed to their perceived effectiveness as workers.

Training contributed to competence and effectiveness. Training, for these participants, tapped into a willingness to be vulnerable for growth. Themes related to hope, use of self in service to others, empathy, and finally, the importance of both boundaries and connections were identified. CPSS' attention was squarely on human relationships and on the needs of their clients. The stories shared by these participants were filled with examples of how they applied their training. Reflected in these stories were core competencies of peer support including engaging in collaborative and caring relationships; offering not simply support but personalized support; sharing lived experiences; providing information; managing crises; valuing communication; engaging in collegial collaboration and teamwork; and promoting growth and development (SAMHSA, 2018). The training and certification processes were important for shaping role expectations and preparing participants for the work. It is understood that in some settings, peer support workers may not be trained or certified. In this case they were, and often the organization paid for this certification. Participants viewed this as an investment in them, and several wanted the organization to get a return on their investment. Given the importance of training in these participants’ perceived effectiveness, it is recommended that national guidelines (SAMHSA, 2023) for peer workers be considered by hiring organizations to safeguard both this workforce and the vulnerable populations with whom they work.

Participants identified the importance of the organization in supporting their effectiveness. Themes related to role clarity, co-worker support along with regular supervision and self-care were identified. Finally, a note was made about the role of organizational leadership in creating and sustaining support for this workforce across the organization. Participants described organizational behaviors and norms in terms of being part of a family. Such norms have the potential to promote shared values, loyalty, and a sense of belonging; on the other hand, this loyalty sometimes leads to workers being exploited by employers for the good of the family (Goffee & Jones, 1998). Miler et al. (2020) cautioned that stigma, workplace experiences of discrimination, and compensation were areas of vulnerability for this workforce. The interview guide contained questions related to these areas. While participants stated that they would appreciate pay raises, stigma and discrimination were not emphasized in what they shared. While workers may have perceived themselves as belonging to an organizational family, another way to frame this is in terms of social innovation. Shier and Handy (2016) suggested that some direct-practice organizations are responding to community needs by creating an organizational culture oriented toward principles of social innovation. They provided a conceptual framework that included “staff engagement: with issues experienced by service users; with the structure or processes involved in decision making in promoting community engagement; with the initiatives undertaken by the organization; with activities that empower staff to act” (p. 127). Such engagement activities were reflected in the participants’ stories and in the way they were encouraged to be authentic and engage in the work of the organization. Miler et al. (2020) proposed that role description, support, training and development, and value were areas to be considered when embedding peer workers in organizations. The participants in this study confirmed this. Workers were empowered to use their skills across the organization and felt supported and valued in doing so. As the organization grows, it is unknown if innovation continues or if it moves through a life cycle to a formalization stage (Lester et al., 2003). At this moment, there seemed to be a good fit between these participants with their lived experiences and unique skill sets, their competencies, and the organization's culture.

### Strengths and Limitations

This study contributes to our understanding of CPSS perceived effectiveness in a community-based organization serving those who experience homelessness. The approach was to assume effectiveness, but questions were posed that identified barriers to providing effective service. The study had some limitations. The first was a small sample size of self-selected participants who may have had more investment in the organization than those who chose to not participate. While we were welcomed into the organization and had key informants collaborating on the design of the study, participants were “hard to reach.” In a similar way that those experiencing homelessness are “hard-to-reach,” recruiting participants with lived experiences of homelessness proved difficult. The second limitation was that data came from self-reports which can invite bias. Chinman et al. (2016) noted that the peer workers in their review rated themselves higher on tasks than supervisors or clients. Finally, independent coding during the analysis phase sought to ensure intercoder reliability. Threats to validity in qualitative research can arise in description, interpretation, or theory (Maxwell, 1992). The debates and discussions that occurred between collaborators during the research process attempted to guard against these threats.

### Implications for Policy, Practice, and Future Research

Given the small size of this study, no generalizations can be made for policy, practice, or research. Some insights are worth considering, however, related to the value of lived and learned knowledge coexisting in organizations serving those who experience homelessness. Studies (National Institute for Care and Excellence [NICE], 2022) are showing that peer workers can be an important part of multidisciplinary teams, delivering care or support and co-designing services. This is useful not only for the services and the people experiencing homelessness but also for the CPSS themselves. The literature (NICE, 2022) highlights the value of involving people with lived experience in the development of policies, procedures, and protocols. CPSS have skills for engaging hard-to-reach populations, given their empathy and commitment. It is important that this workforce not be exploited due to their willingness to go the extra mile for clients. Training and certification in preparation for the role seem useful to help channel CPSS experiences towards competence. Supervision is needed to ensure ongoing competency and advancement, and to provide an off-ramp as needed. We see this as a pilot study worthy of replication. Future research might ask the clients of CPSS directly about their effectiveness. As former clients themselves, some of the CPSS we spoke with confirmed the value of those who had “been there” in their own journeys out of homelessness.

## Footnotes

1 Peer workers are variously called peer supporters, peer support specialists, peer providers, peer navigators, etc. Peer workers can apply for certification and undergo training. A national model for peer work offers guidance to promote quality for mental health certification, substance use certification, and family peer certification (Substance Abuse and Mental Health Services Administration, 2023).

2 Certification requirements vary by state. For a comparison, see Peer Recovery Center of Excellence (2023)
